# A predictive nomogram for lymph node metastasis of incidental gallbladder cancer: a SEER population-based study

**DOI:** 10.1186/s12885-020-07341-y

**Published:** 2020-08-31

**Authors:** Yingnan Yang, Zhuolong Tu, Huajie Cai, Bingren Hu, Chentao Ye, Jinfu Tu

**Affiliations:** Department of Hepatobiliary Surgery, First Affiliated Hospital of Wenzhou Medical University, Nanbaixiang street, Ouhai district, Wenzhou, Zhejiang Province China

**Keywords:** SEER, Incidental gallbladder cancer, Nomogram, Lymph node metastasis, Predict

## Abstract

**Background:**

Existing imaging techniques have a low ability to detect lymph node metastasis (LNM) of gallbladder cancer (GBC). Gallbladder removal by laparoscopic cholecystectomy can provide pathological information regarding the tumor itself for incidental gallbladder cancer (IGBC). The purpose of this study was to identify the risk factors associated with LNM of IGBC and to establish a nomogram to improve the ability to predict the risk of LNM for IGBC.

**Methods:**

A total of 796 patients diagnosed with stage T1/2 GBC between 2004 and 2015 who underwent surgery and lymph node evaluation were enrolled in this study. We randomly divided the dataset into a training set (70%) and a validation set (30%). A logistic regression model was used to construct the nomogram in the training set and then was verified in the validation set. Nomogram performance was quantified with respect to discrimination and calibration.

**Results:**

The rates of LNM in T1a, T1b and T2 patients were 7, 11.1 and 44.3%, respectively. Tumor diameter, T stage, and tumor differentiation were independent factors affecting LNM. The C-index and AUC of the training set were 0.718 (95% CI, 0.676–0.760) and 0.702 (95% CI, 0.659–0.702), respectively, demonstrating good prediction performance. The calibration curves showed perfect agreement between the nomogram predictions and actual observations. Decision curve analysis showed that the LNM nomogram was clinically useful when the risk was decided at a possibility threshold of 2–63%. The C-index and AUC of the validation set were 0.73 (95% CI: 0.665–0.795) and 0.692 (95% CI: 0.625–0.759), respectively.

**Conclusion:**

The nomogram established in this study has good prediction ability. For patients with IGBC requiring re-resection, the model can effectively predict the risk of LNM and make up for the inaccuracy of imaging.

## Backgroud

Gallbladder cancer (GBC) is a rare malignancy. Its annual incidence rate is 2.2 per 100,000 [1]. GBC has no obvious characteristic manifestations at its early stage, so it is difficult to identify early. However, it is highly invasive [2]. Most GBC patients are at an advanced stage once confirmed and lose the opportunity for surgical treatment [3]. Fortunately, with the development of laparoscopy, an increasing number of GBCs may be confirmed at the early stage through laparoscopic cholecystectomy (LC) so that early R0 resection may be performed; thus, progression of the disease may be avoided, and the overall survival rate may be improved [4, 5].

More than 50% of GBCs are diagnosed by intraoperative or postoperative pathological examination after LC [4] and are considered incidental gallbladder cancer (IGBC), in which stage T1/2 GBCs are the most common [6]. IGBC often requires radical re-resection [5]. Among patients with lymph node metastasis (LNM), lymph node dissection is an important part of radical surgery [7]. Although an increasing number of clinical centers emphasize the importance of high-quality lymph node dissection [8–10], a study based on the SEER database showed that the lymph node resection rates for stages T1a, T1b, and T2 GBC were only 33.6, 39.2, and 53.7%, respectively [7], which indicated that preoperative lymph node examination was seriously insufficient. LNM is an independent factor influencing the prognosis of early GBC [11, 12]. Therefore, the preoperative diagnosis of LNM is very important. However, current imaging is still not sensitive enough to identify LNM in the preoperative examination [13]. In lieu of the low incidence rate of GBC, there is still no study with a large sample size for predicting the risk factors for LNM in early GBC, and there is no quantified prediction model.

LC makes general pathological information on patients with IGBC available before the patients receive re-resection [2]. In recent years, nomograms have been broadly used for preoperative prediction of the risk of LNM and have been proven to be effective [14–16]. Therefore, this study aims to use the pathological and demographic information contained in the SEER database to determine the LNM risk factors for IGBC and to establish a nomogram model for predicting the incidence rate of LNM at the early stage of IGBC before re-resection.

## Methods

### Data collection

The SEER (Surveillance, Epidemiology, and End results) database is currently the largest publicly available cancer database, covering approximately 28% of the US population [3]. The National Cancer Institute’s SEER*Stat software (8.3.6 version) was used to collect data. The inclusion criteria were as follows: (1) site record: C23.9, according to the Third Edition of International Classification of Diseases for Oncology (ICD-O-3); (2) pathological type: adenocarcinoma or squamous cell carcinoma; (3) T stage classified as T1a, T1b, T2 and N stage classified as N0 and N1 according to 6th edition AJCC staging system; (4) underwent surgery; (5) at least 1 regional lymph node examined; and (6) no preoperative radiotherapy. After the inclusion, patients were excluded if their information regarding tumor size or tumor differentiation was unknown. We also excluded patients diagnosed with M1 stage, for whom surgery was not suitable [17].

We extracted the demographic and clinicopathologic data of patients with T1/2 GBC from the SEER database for model development and validation, including age, sex, race, tumor size, histology, differentiation, depth of invasion, and number of lymph nodes examined.

The whole dataset from the SEER database was randomly partitioned into a training set and a validation set, which included 70 and 30% of the dataset, respectively. To let each data has the same chance to be assigned to training set and validation set, a simple random sampling method was used for allocation. Specifically, we installed caret package in R software version 3.6.2, then we loaded the foreign, survival and caret packages. And the last step was to run the packages by specific codes. The codes were attached in our Supplementary Material.

### Statistical analysis

Correlations between the clinicopathological characteristics of patients and LNM were analyzed using Pearson’s chi-square test or Fisher exact test when needed. To identify factors that were associated with LNM, binary logistic regression analysis was used for univariate and multivariable analyses. Odds ratios (ORs) were presented with 95% CIs. Preoperatively available variables were included in the logistic regression analysis. To construct a well-calibrated and discriminative nomogram for predicting LNM, a model was developed in a training set and then validated in the validation set. A logistic regression model was used to construct the nomogram with a backward stepwise procedure. Variables with *P* < 0.05 were included in the nomogram.

Nomogram performance was quantified with respect to discrimination and calibration. Discrimination (the ability of a nomogram to separate patients with different lymph node statuses) was quantified by concordance indexes (C-indexes) and the area under the receiver operating characteristic (ROC) curve (AUC). Calibration was assessed graphically by plotting the relationship between the actual (observed) probabilities and predicted probabilities (calibration plot) with the bootstrapping method (1000 replications). Clinical usefulness and net benefit were estimated with decision curve analysis (DCA).

Statistical analyses of correlations between clinicopathological characteristics were conducted using SPSS version 24.0 (IBM, NY, US). The partition of dataset, logistic regression analysis, construction and performance quantification of nomogram and DCA were conducted using R statistical software version 3.6.2. All tests were two-sided, and *P* < 0.05 was deemed significant.

## Results

### Demographics and pathological characteristics

Table 1 summarizes in detail the clinicopathological characteristics of 796 patients diagnosed with stage T1/2 GBC between 2004 and 2015. The LNM rates of T1a, T1b and T2 GBC in the total population were 7, 11.1 and 44.3%, respectively. There were 560 patients in the training set: 205 patients with LNM(+) and 355 patients with LNM(−). There were 236 patients in the validation set: 88 patients with LNM(+) and 148 patients with LNM(−). The degree of tumor differentiation, T staging and tumor diameter were all associated with LNM in both groups (*P* < 0.05). The median number of lymph nodes examined in training set was 2 (IQR: 1–5).

**Table 1:** Correlations between clinicopathological characteristics of patients and LNM in the training and validation sets

Characteristics	Training Cohort			Validation set		
	LNM-	LNM+	*P* value	LNM-	LNM+	P value
Median number of retrieved LN (IQR)	2 (1–5)		/	2 (1–4)		/
Age						
≤ 60	103 (29.0%)	54 (26.3%)	0.561	40 (27.0%)	21 (23.9%)	0.702
>60	252 (71.0%)	151 (73.7%)		108 (73.0%)	67 (76.1%)	
Gender						
Male	112 (31.5%)	52 (25.3%)	0.121	46 (31.1%)	18 (20.5%)	0.076
Female	243 (68.5%)	153 (74.6%)		102 (68.9%)	70 (79.5%)	
Race						
White	249 (70.1%)	157 (76.6%)	0.214	108 (73.0%)	66 (75.0%)	0.456
Black	50 (14.1%)	20 (9.8%)		19 (12.8%)	14 (15.9%)	
Others	56 (15.8%)	28 (13.7%)		21 (14.2%)	8 (9.1%)	
Histology						
Adenocarcinoma	346 (97.5%)	203 (99.0%)	0.343*	147 (99.3%)	88 (100.0%)	0.999*
Squamous cell carcinoma	9 (2.5%)	2 (1.0%)		1 (0.7%)	0 (0.0%)	
Grade						
Well differentiated	87 (24.5%)	26 (12.7%)	< 0.001	39 (26.4%)	11 (12.5%)	0.008
Moderately differentiated	181 (51.0%)	96 (46.8%)		71 (48.0%)	40 (45.5%)	
Poorly/un- differentiated	87 (24.5%)	83 (40.5%)		38 (25.7%)	37 (42.0%)	
T stage						
T1a	38 (10.7%)	2 (1.0%)	< 0.001	15 (10.1%)	2 (2.3%)	< 0.001
T1b	75 (21.1%)	8 (3.9%)		29 (19.6%)	5 (5.7%)	
T2	242 (68.2%)	195 (95.1%)		104 (70.3%)	81 (92.0%)	
Tumor size						
≤ 1 cm	67 (18.9%)	10 (4.9%)	< 0.001	34 (23.0%)	5 (5.7%)	0.005
>1 cm	288 (81.1%)	195 (95.1%)		114 (77.0%)	21 (94.3%)	

LNM lymph node metastasis; IQR interquartile rage; * *P* value is derived from Fisher’s exact test; other *P* values are derived from Pearson’s chi-square test

### Factors associated with preoperative LNM

As shown in Table 2, the logistic regression model was used to further verify the effectiveness of the included factors. Univariate analysis showed that tumors with a diameter > 1 cm, stage T2, and poor/undifferentiation were closely related to LNM. Multivariate analysis further confirmed that tumors with a diameter > 1 cm (OR = 3.628, 95% CI: 1.770–7.437), stage T2 (OR = 11.104, 95% CI: 2.590–47.597), and poor/undifferentiation (OR = 2.110, 95% CI: 1.184–3.762) were independent factors influencing LNM. Based on the OR value, T2 stage was the most correlated, followed by the tumor diameter and then the degree of differentiation. Age, sex, race and pathological pattern were not significantly correlated with LNM.

**Table 2:** Logistic regression analysis of risk factors for LNM in training cohort

Variable	Univariate snalysis		Multivariate analysis	
	Crude OR(95%CI)	P value	Ajusted OR(95%CI)	P value
Age				
≤ 60	1.00(reference)			
>60	1.143 (0.777–1.682)	0.498		
Gender				
Male	1.00(reference)			
Female	1.356 (0.922–1.995)	0.122		
Race				
White	1.00(reference)			
Black	0.634 (0.364–1.106)	0.108		
Others	0.793 (0.483–1.302)	0.359		
Histology				
Adenocarcinoma	1.00(reference)			
Squamous cell carcinoma	0.379 (0.081–1.770)	0.217		
Grade				
Well differentiated	1.00(reference)		1.00(reference)	
Moderately differentiated	1.775 (1.073–2.935)	0.025	1.260 (0.730–2.177)	0.407
Poorly/un- differentiated	3.192 (1.876–5.431)	< 0.001	2.110 (1.184–3.762)	0.011
T stage				
T1a	1.00(reference)		1.00(reference)	
T1b	2.027 (0.410–10.017)	0.386	1.595 (0.316–8.058)	0.572
T2	15.31 (3.648–64.255)	< 0.001	11.104 (2.590–47.597)	< 0.001
Tumor size				
≤ 1 cm	1.00(reference)		1.00(reference)	
>1 cm	4.536 (2.278–9.034)	< 0.001	3.628 (1.770–7.437)	< 0.001

LNM lymph node metastasis; OR odds ratio; 95%CI 95% confidence interval

### Nomogram development

**Fig. 1:**
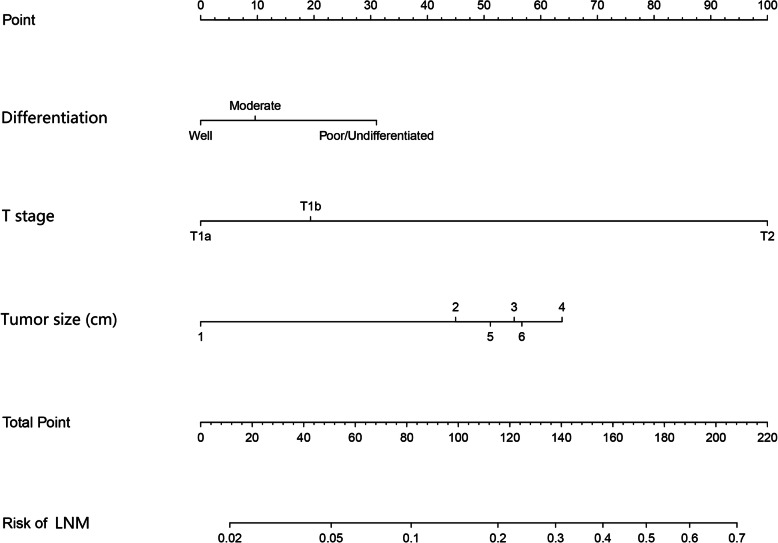
Nomogram for predicting LNM in patients with T1/T2 gallbladder cancer. To use the nomogram, a factor’s value of an individual patient was located on each axis, and a line was drawn upward to determine the points received for each variable value. The points for each variable were summed and located on the total point line. And then, the bottom line corresponding vertically to the above total line illustrated the individual predictive risk for LNM. (LNM, lymph node metastasis)

Logistic regression indicated that tumor diameter, T stage and differentiation degree were independent factors influencing LNM. We included these three variables and constructed a nomogram (Fig. 1). To predict the risk of LNM in more detail, we further subdivided the tumor diameters as follows: 1 = “d ≤ 1 cm”, 2= “d≤ 2 cm”, 3= “d≤ 3 cm”, 4= “d≤ 4 cm”, 5= “d≤ 5 cm”, and 6= “d> 5 cm”.

### Validation of the model

**Fig. 2:**
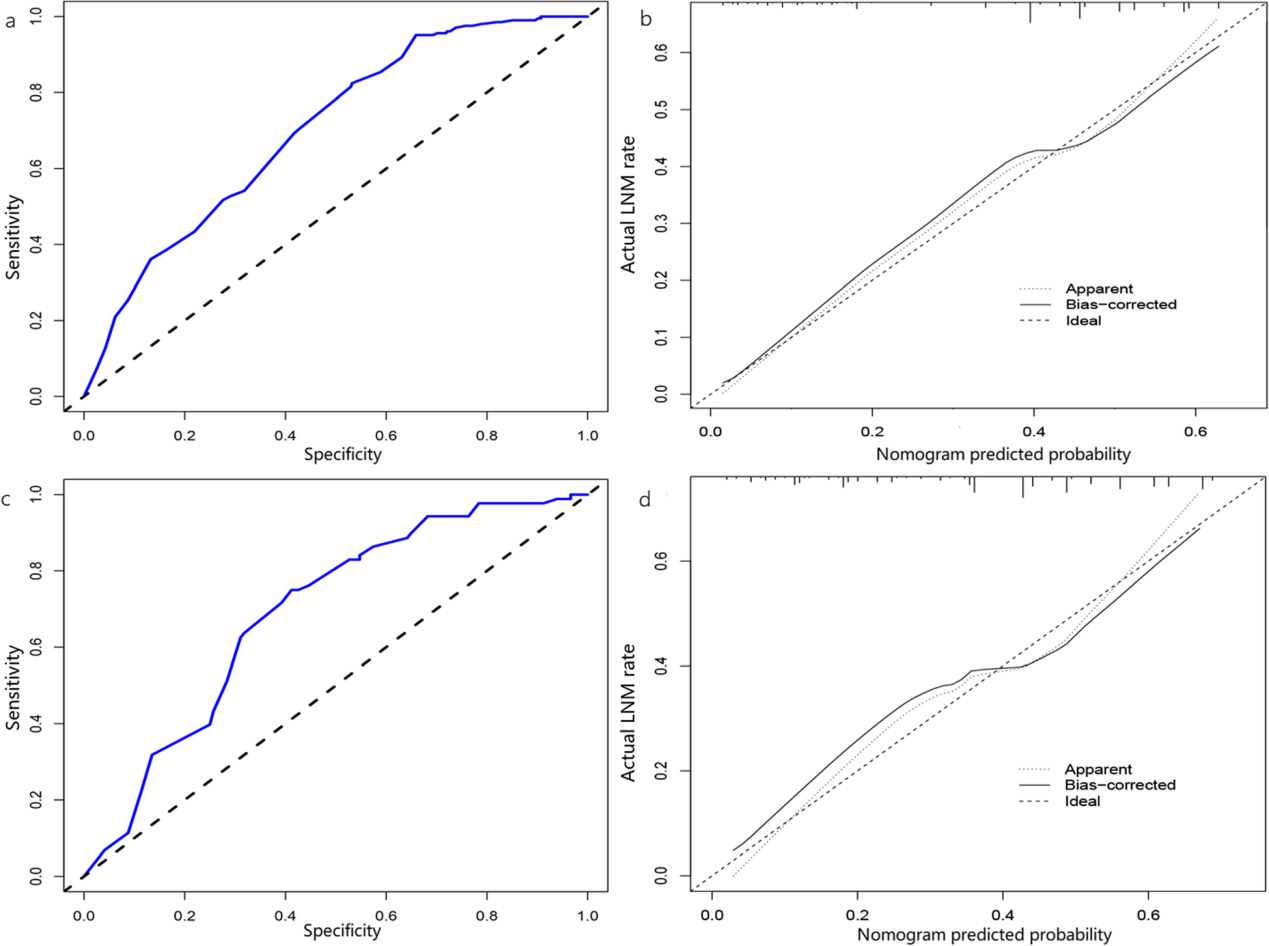
Discrimination and validation of nomogram for predicting LNM in T1/T2 gallbladder cancer. **a** and **c** ROC for discrimination in the training and validation sets. The AUCs of the nomograms were 0.702 (95% CI 0.659–0.745) and 0.692 (95% CI 0.625–759), respectively. **b** and **d** Calibration plot for the nomogram in the training and validation sets. The x-axis represents the nomogram predicted probabilities as measured by logistic regression analysis, and the y-axis represents the actual probabilities. (ROC, receiver operating characteristics; AUC, area under the curve; 95%CI, 95% confidence interval)

The nomogram demonstrated good accuracy for predicting positive lymph nodes, with a C-index of 0.718 (95% CI, 0.676–0.760) and an AUC of 0.702 (95% CI, 0.659–0.702). The calibration plot presented good agreement between the bias-corrected prediction and the ideal reference line with an additional 1000 bootstraps (mean absolute error = 0.02) (Fig. 2a, c).

The C-index and AUC of the validation set were 0.73 (95% CI: 0.665–0.795) and 0.692 (95% CI: 0.625–0.759), respectively, which revealed good concordance and reliable ability to estimate the status of lymph node involvement. The calibration plot of validation also demonstrated good agreement between the bias-corrected prediction and the ideal reference line with an additional 1000 bootstraps (mean absolute error = 0.035) (Fig. 2b, d).

### Comparison between different prediction methods

**Fig. 3:**
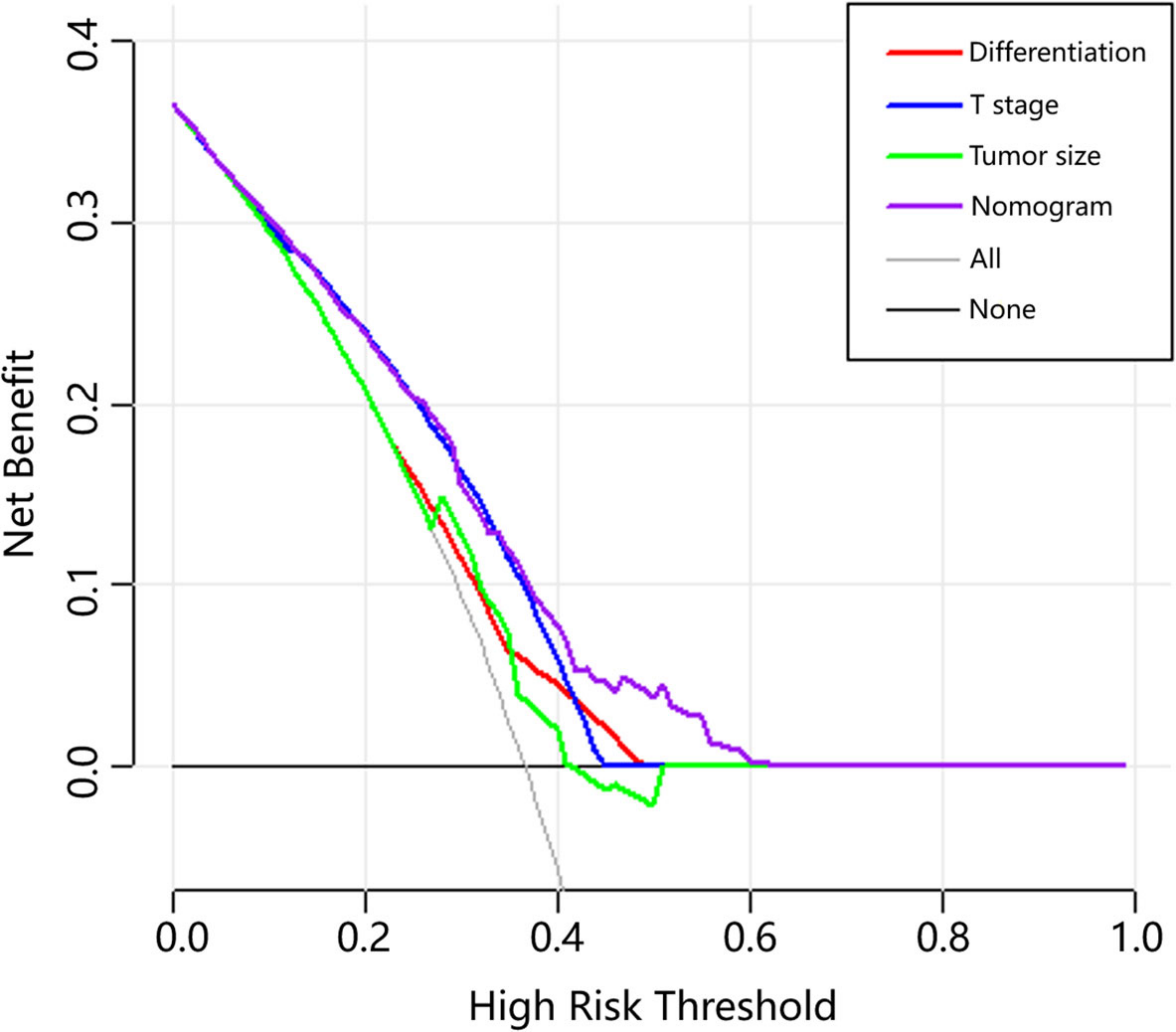
Decision curve for prediction of LNM for T1/T2 gallbladder cancer. Black line: assume no patient will have LNM; gray line: assume all patients will have LNM; red line: binary decision rule based on tumor differentiation alone; blue line: binary decision rule based on T stage alone; green line: binary decision rule based on tumor size alone; purple line: decision based on nomogram. Probability thresholds for differentiation, T stage, tumor size and nomogram are 0.23–0.49, 0.03–0.45, 0.28–0.51, 0.02–0.63, respectively

Comparisons between different prediction methods were conducted by decision curve analysis. The decision curve has the ability to show the clinical usefulness of each method based on a continuum of potential thresholds for LNM risk (x-axis) and the net benefit of using the model to risk stratify patients (y-axis) relative to assuming that no patient will have LNM. Figure 3 reveals that the nomogram provided the largest net benefit across the range of LNM risk compared with the methods using tumor size, differentiation and T-stage alone.

## Discussion

GBC is a highly occult cancer with no obvious clinical manifestations in its early stage [3]. With the development of laparoscopy, an increasing number of stage T1/2 IGBCs can be detected via pathological biopsy after LC [6]. For IGBCs, postoperative pathological evaluations need to be completed in combination with imaging for re-resection [18, 19].

For patients with LNM, lymphadenectomy is an important part of radical resection, and all positive lymph nodes need to be cleared [20]. Although high-quality lymph node dissection was emphasized, preoperative lymph node examination was seriously insufficient based on the results that the resection rates of T1a, T1b, and T2 GBC were only 33.6, 39.2, and 53.7%, respectively, according to this SEER-based study [7]. Although current NCCN guidelines recommend radical surgery for all patients with GBC at stages T1b and above [18], several studies have concluded that patients with T1b and T2 stages might not require radical surgery [21–24]. However, some studies have shown that LNM is closely related to malignant phenotype of early stage GBC [25, 26], we believe that patients diagnosed with LNM preoperatively should receive more aggressive surgical treatment and more extensive lymph node dissection than patients without LNM.

CT is the most commonly used clinical imaging method [27]. Although CT can accurately show the invasion of tumors in blood vessels and adjacent organs, its accuracy for the identification of LNM is very low [28]. Some studies have shown that more than half of the positive lymph nodes existing among GBC patients cannot be detected by preoperative CT examination [24, 27, 29]. Unfortunately, neither MRI nor PET-CT is a good supplement for CT [28, 30, 31]. The present study may combine clinical imaging to further improve the estimation of the risk of LNM, which is conducive to clinicians choosing the most suitable surgical methods for patients.

Among the cases of GBC included in this study, the LNM rate of stage T1a was 7%, stage T1b was 11.1%, and stage T2 was 44.3%. For a variety of early primary cancers in the digestive tract, such as gastric cancer [14], appendiceal cancer [15], and colon cancer [16], the SEER database has been used to establish a nomogram for predicting the risk of LNM. In this paper, the SEER database was used to predict the risk of LNM in IGBC and construct a nomogram. In the present study, tumor diameter, tumor differentiation degree and T stage were independent factors influencing metastasis, of which T stage was the most significant factor. Compared with that at stage T1a, the risk of LNM at stage T2 may have increased by 11 times. The second most significant factor was tumor diameter. When the tumor diameter was greater than 1 cm, the risk of LNM may have increased by 3.6 times. According to the nomogram, there was little difference in the risk of LNM when the tumor diameter was greater than 1 cm, but the risk was reduced when the tumor diameter was greater than 4 cm. The least significant factor was tumor differentiation. The risk of LNM in poorly differentiated or undifferentiated patients was only twice as high as that in well-differentiated patients. Gallbladder adenocarcinoma (76–90%) and squamous cell carcinoma (2–10%) are the two most common pathological patterns of GBC and the prognosis of squamous cell carcinoma is worse than that of adenocarcinoma [32], but in our study, it is indicated that there was no significant correlation between pathological patterns and LNM. We believe that there are two possibilities: (1) according to the relevant literature, squamous cell carcinoma is more likely to invade the liver than LNM [33], which may further confirm that there is no correlation pathological patterns in LNM; and (2) the number of T1/2 squamous cell carcinomas is too small to be statistically significant.

Considering the low incidence rate of GBC, few single-center studies have previously used clinical data to predict the risk of LNM in early GBC. Therefore, we used DCA to compare the differences in predictive power among the nomogram and the included univariates. According to Fig. 3, the probability thresholds of differentiation, T stage, tumor size and nomogram are 0.23–0.49, 0.03–0.45, 0.28–0.51 and 0.02–0.63, respectively. The curve of T-stage is very close to that of nomogram containing three factors, but the probability threshold of T-stage is smaller than that of nomogram. When the risk is decided at a probability threshold lower than 0.38, the T-stage curve and the nomogram curve almost overlap which indicates the two prediction models almost have the same net benefit within this range, and both are higher than the reference line. However, when the risk is decided higher than 0.38, the net benefit of T-stage is not as good as that of the nomogram. A comparison between tumor and differentiation shows that when the risk is decided at a probability threshold of 0.23–0.28, the net benefits of tumor and differentiation are very close and nearly equal to the reference line; when the probability is decided at a probability threshold of 0.28–0.35, the net benefits of these two are still very close, but higher than the reference line; when the risk is decided at a probability threshold of 0.35 and 0.4, the net benefit of differentiation is relatively high; and when the probability is decided higher than 0.4, the net benefit of tumor size is less than 0 while the differentiation model has a prediction ability higher than that of the tumor model. However, the net benefits of these two models within their probability thresholds are both smaller than that of the nomogram. To sum up, although the univariate models have certain predictive power, DCA shows that the nomogram predicts accurately in a wider range.

For GBC patients accompanied by LNM, existing studies recommend cholecystectomy and lymph node dissection for patients at stage T1a [34], and radical surgery for patients at stage T1b/T2 [26]. The total score calculated by the nomogram corresponds to the risk of LNM. Zhu et al. [35] put forward that patients with a ≤ 5.0% predicted risk of LNM are considered as low-risk group, those with 5–15% predicted risk as intermediate risk group, and those predicted risk >15% as high risk group. Combining these conclusions with our study, we assume that patients in low-risk group could choose long-term follow-up, and patients in the high-risk group should be recommended for a re-resection; as for those in intermediate-risk group, patients could choose a long-term follow-up, however, the recommendation of re-resection should better be come up with. Take a T1b IGBC patients for example, in clinical practice, if a T1b IGBC patient pathologically diagnosed after LC is with poor compliance to a re-resection, in the meanwhile, no LNM is found by imaging, which is considered having low ability to detect LNM [27, 28, 30, 31], the clinician will be caught into a dilemma that whether a re-resection is needed or not. In this case, the clinician may use our nomogram to make a decision. If he/she is pathologically confirmed with a poorly differentiated or undifferentiated tumor with a diameter between 3 and 4 cm, his/her total score will be 113. His/her corresponding risk of LNM is nearly 19% and is allocated to high-risk group. The clinical suggestion is that him/her should undergo a radical re-resection. In contrast, if the T1b patient is with a highly differentiated tumor with a diameter less than 1 cm, his/her total score will be 20, and the risk of LNM is nearly 3% and is allocated to low-risk group. The clinical suggestion is that he/she could choose to follow up regularly.

We must recognize the limitations that may exist with our study. First, all selected patients have received lymph nodes biopsy and the median number of lymph nodes inspected in training set was 2 (IQR: 1–5), however, the effect of selection bias with LN+ and LN- due to the non-randomized nature of this study can’t be expected. Steffen et al. [7] claimed that retrieval of even a few lymph nodes reliably predicts the lymph node status, which may compensate for this bias. Second, previous studies have concluded that age < 60, elevated CA199 levels [27], and hepatic-sided tumors [36] can also be used for predicting LNM. However, in this study, age was not necessarily associated with LNM, and this study lacked information concerning the preoperative diagnosis of CA199 and tumor location, which may have led to insufficient influencing factors. Last but not least, the data in SEER database is originated from different sources and hospitals [3], so our study is considered as a multicenter study. However, GBC has regional differences in incidence [37]. Although the nomogram constructed in this study was validated internally and externally having good prediction ability, in our view, the generalization ability of the nomogram is still needed to be verified with clinical data other than SEER database. Therefore, we hope that in the future, large sample of GBC patients from different regions can be obtained to construct a nomogram using the three variables selected in this study for further external validation, as well as measurement of the generalization ability of the nomogram.

Despite limitations above, the large-sample based study predicts LNM with good discrimination and calibration both in the training and validation cohorts. The nomogram constructed in this study visualizes the risk factors and could better guide the clinical decisions.

## Conclusion

In conclusion, based on the clinical risk factors identified in a large population-based cohort, we established the first practical nomograms that could objectively and accurately predict the individualized risk of LNM for IGBC patients who required re-resection. Moreover, the validation set results demonstrated that the nomograms performed well and had high accuracy and reliability. Our nomogram was demonstrated to be clinically useful in DCA, and it made up for the inaccuracy of imaging.

Therefore, these results could help clinicians improve individual treatment and make clinical decisions regarding patients with T1/2 stage IGBC.

## Supplementary information

**Additional file 1.**

## Data Availability

The datasets analyzed during the current study are available in the SEER repository (https://seer.cancer.gov/).

## Abbreviations

AJCC: American Joint Committee on Cancer
AUC: area under the receiver operating characteristic curve;
C-indexes: concordance indexes
CI: confidence intervals
DCA: decision curve analysis
GBC: gallbladder cancer
IGBC: incidental gallbladder cancer
IQR: interquartile rage
LC: laparoscopic cholecystectomy
LNM: lymph node metastasis
OR: odds ratio
ROC: receiver operating characteristic
SEER: Surveillance, Epidemiology, and End results
