# Supplementary material for: A predictive nomogram for lymph node metastasis of incidental gallbladder cancer: a SEER population-based study
Source: BMC Cancer. 2020 Aug 31;20:828. doi: 10.1186/s12885-020-07341-y (PMC7461264; doi:10.1186/s12885-020-07341-y)
Supplement: Supplementary file 1 — Additional file 1. [file 12885_2020_7341_MOESM1_ESM.docx]

install.packages("caret")

library(foreign)

library(survival)

library(caret)

setwd("C:\\Users\\Desktop\\logistics\\BMC cancer")

non_tumor<-read.table("input.txt",header=T,sep="\t")

set.seed(300)

non_tumord<-createDataPartition(y=non_tumor$id,p=0.70,list=F)

non_tumordev<-non_tumor[non_tumord, ]

non_tumorv<-non_tumor[-non_tumord,]

write.csv(non_tumordev, "non_tumordev.csv")

write.csv(non_tumorv, "non_tumorv.csv")
